# NRF2 Is a Key Target for Prevention of Noise-Induced Hearing Loss by Reducing Oxidative Damage of Cochlea

**DOI:** 10.1038/srep19329

**Published:** 2016-01-18

**Authors:** Yohei Honkura, Hirotaka Matsuo, Shohei Murakami, Masayuki Sakiyama, Kunio Mizutari, Akihiro Shiotani, Masayuki Yamamoto, Ichiro Morita, Nariyoshi Shinomiya, Tetsuaki Kawase, Yukio Katori, Hozumi Motohashi

**Affiliations:** 1 Department of Otolaryngology-Head & Neck Surgery, Tohoku University Graduate School of Medicine, 1-1 Seiryo-machi, Aoba-ku, Sendai, 980-8574, Japan; 2 Department of Gene Expression Regulation, Institute of Development, Aging and Cancer, Tohoku University, 4-1 Seiryo-machi, Aoba-ku, Sendai, 980-8575, Japan; 3 Department of Integrative Physiology and Bio-Nano Medicine, National Defense Medical College, 3-2 Namiki, Tokorozawa, Saitama 359-8513, Japan; 4 Department of Otolaryngology-Head and Neck Surgery, National Defense Medical College, 3-2 Namiki, Tokorozawa, Saitama 359-8513, Japan; 5 Department of Medical Biochemistry, Tohoku University Graduate School of Medicine, 2-1 Seiryo-machi, Aoba-ku, Sendai, 980-8575, Japan; 6 Department of Otolaryngology-Head and Neck Surgery, Self-Defense Forces Central Hospital, 1-2-24, Ikejiri, Setagaya, Tokyo 154-8532, Japan

## Abstract

Noise-induced hearing loss (NIHL) is one of the most common sensorineural hearing deficits. Recent studies have demonstrated that the pathogenesis of NIHL is closely related to ischemia-reperfusion injury of cochlea, which is caused by blood flow decrease and free radical production due to excessive noise. This suggests that protecting the cochlea from oxidative stress is an effective therapeutic approach for NIHL. NRF2 is a transcriptional activator playing an essential role in the defense mechanism against oxidative stress. To clarify the contribution of NRF2 to cochlear protection, we examined *Nrf2*^–/–^ mice for susceptibility to NIHL. Threshold shifts of the auditory brainstem response at 7 days post-exposure were significantly larger in *Nrf2*^–/–^ mice than wild-type mice. Treatment with CDDO-Im, a potent NRF2-activating drug, before but not after the noise exposure preserved the integrity of hair cells and improved post-exposure hearing levels in wild-type mice, but not in *Nrf2*^–/–^ mice. Therefore, NRF2 activation is effective for NIHL prevention. Consistently, a human *NRF2* SNP was significantly associated with impaired sensorineural hearing levels in a cohort subjected to occupational noise exposure. Thus, high NRF2 activity is advantageous for cochlear protection from noise-induced injury, and NRF2 is a promising target for NIHL prevention.

Noise-induced hearing loss (NIHL) and age-related hearing loss (AHL) are two major classes of sensorineural hearing loss (SNHL) in adult populations. Recent studies have demonstrated that excessive oxidative stress in the cochlea is closely related to the pathogenesis of both NIHL and AHL, implying that appropriate control of oxidative stress is an effective strategy to prevent the development and progression of the majority of SNHL cases in adulthood. How to strengthen cochlear resistance against oxidative stress is an important issue for maintenance of the structural and functional integrity of the cochlea. In particular, NIHL is caused by prolonged and/or repeated exposure to excessive noise in occupational or recreational situations, most of which are predictable and should thus be preventable. The reinforcement of defense mechanisms against oxidative stress before the noise exposure is expected to be a promising preventive strategy for people who are inevitably exposed to scheduled noise. Currently, however, no drugs have been launched for the purpose of NIHL prevention.

During noise exposure, blood circulation is impaired in the cochlea due to the constriction of blood vessels^1^,^2^,^3^,^4^,^5^. An increase of 8-isoprostane-F2a (a potent vasoconstrictor) in the cochlea during noise exposure has been suggested to be responsible for the vasoconstriction^6^,^7^. After noise exposure, cochlear blood flow gradually recovers^8^, which provokes ischemia-reperfusion injury in the cochlea. Mitochondrial dysfunction develops during ischemia-reperfusion injury in the heart, lung, liver and kidney, resulting in increased production of reactive oxygen species (ROS) and tissue damage^9^,^10^. ROS levels are also elevated in the cochlea after noise exposure, and antioxidant reagents have been shown to protect the cochlea from noise-induced damage^3^.

The transcription factor NRF2 is a master regulator of numerous detoxifying and antioxidant genes and is activated in response to oxidative and electrophilic stress^11^,^12^. The most unique feature of NRF2 is its induction by stress. In normal conditions, NRF2 is ubiquitinated by the KEAP1-CULLIN3 ubiquitin E3 ligase complex in the cytoplasm, resulting in degradation through the proteasome. Upon exposure to ROS or electrophiles, which inactivate KEAP1, NRF2 is stabilized, translocates into the nucleus and activates many cytoprotective genes, which confers resistance to the oxidative and electrophilic stress. Miscellaneous NRF2-activating reagents have been identified and applied to ischemia-reperfusion injury in animal experiments, with successful improvement of the pathological states in the majority of cases^13^,^14^,^15^. However, the contribution of NRF2 activation to cochlear protection from excessive noise has not been clarified.

To elucidate the contribution of NRF2 to cochlear protection from noise-induced damage, we performed noise exposure experiments using *Nrf2*^–/–^ mice. *Nrf2*^–/–^ mice exhibited more severe impairment of hearing levels than wild-type mice at 7 days post-exposure, whereas pre-activation of NRF2 by 2-cyano-3,12 dioxooleana-1,9 dien-28-imidazolide (CDDO-Im) dramatically improved the post-exposure hearing levels in wild-type mice but not in *Nrf2*^–/–^ mice. CDDO-Im is a highly potent NRF2 inducer that has been well tested *in vivo*^16^,^17^,^18^. Consistent with the results obtained in the mouse experiments, a single nucleotide polymorphism (SNP) in the *NRF2* promoter, which reduces the transcription level of the *NRF2* gene, was significantly associated with impaired sensorineural hearing levels in a human cohort that was subjected to occupational noise exposure. These results demonstrated that high NRF2 activity is beneficial for cochlear protection from noise-induced injury and suggested that NRF2 is a key target for the prevention of NIHL.

## Results

### NRF2 deficiency exacerbates NIHL

**Figure 1:**
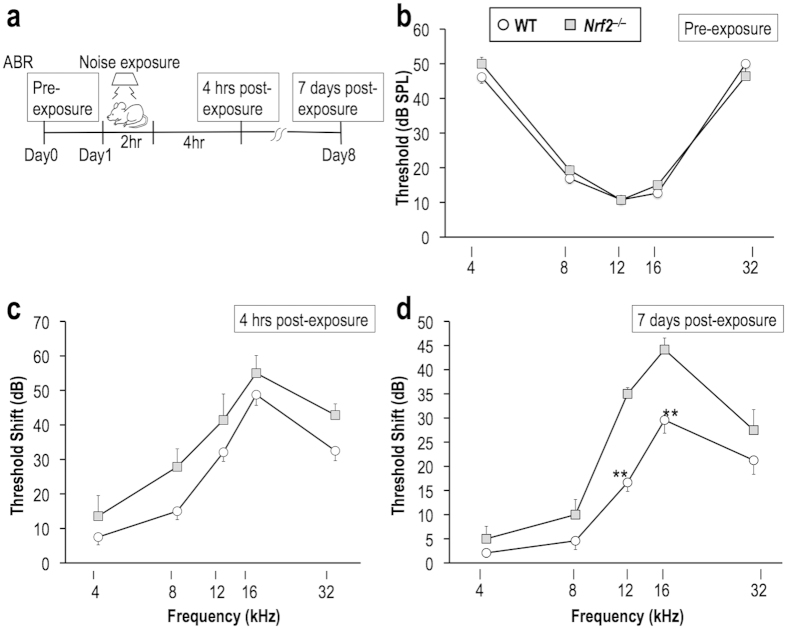
NRF2 deficiency increases susceptibility to noise exposure. (**a**) Experimental design for acoustic overexposure and ABR test. Wild-type and *Nrf2*^*–/–*^ mice were exposed to 96-dB noise for 2 hr. ABR thresholds were measured for one ear at 4, 8, 12, 16, and 32 kHz, 1 day before, 4 hr after, and 7 days after noise exposure. (**b**) ABR thresholds for each test frequency before noise exposure. (**c,d**) ABR threshold shifts at 4 hr (**c**) and 7 days (**d**) after noise exposure. **P < 0.01. Unpaired two-tailed Student’s *t*-test was applied. The data represent the mean ± s.e.m. (n = 7–8).

To address whether NRF2 deficiency causes susceptibility to NIHL, we conducted noise-exposure experiments followed by auditory brainstem response (ABR) recording using wild-type and *Nrf2*^–/–^ mice. Mice were exposed to 96-dB noise continuously for 2 hr, and ABR thresholds were measured for one ear at 4, 8, 12, 16, and 32 kHz 1 day before and then 4 hr and 7 days after the noise exposure (Fig. 1a). The ABR thresholds before the noise exposure were almost comparable between the genotypes (Fig. 1b), indicating that NRF2 is not essential for the normal development and function of the cochlea. ABR threshold shifts at 4 hr post-exposure (temporary threshold shift; TTS) were not significantly different between the genotypes (Fig. 1c). In contrast, ABR threshold shifts at 7 days post-exposure (permanent threshold shift; PTS) were significantly larger in the *Nrf2*^–/–^ mice than in the wild-type mice (Fig. 1d), indicating that the *Nrf2*^–/–^ mice are more susceptible to noise exposure and that recovery from the TTS is impaired in the absence of NRF2.

### Noise exposure does not make obvious changes in NRF2 target gene expression

Acoustic overexposure has been reported to cause ischemia-reperfusion and to increase the production of reactive oxygen species (ROS) and reactive nitrogen species (RNS), including hydrogen peroxide, superoxide and peroxynitrite. Because NRF2 has been shown to induce a battery of cytoprotective genes in response to ROS/RNS, we expected that NRF2 target genes would be activated after noise exposure.

**Figure 2:**
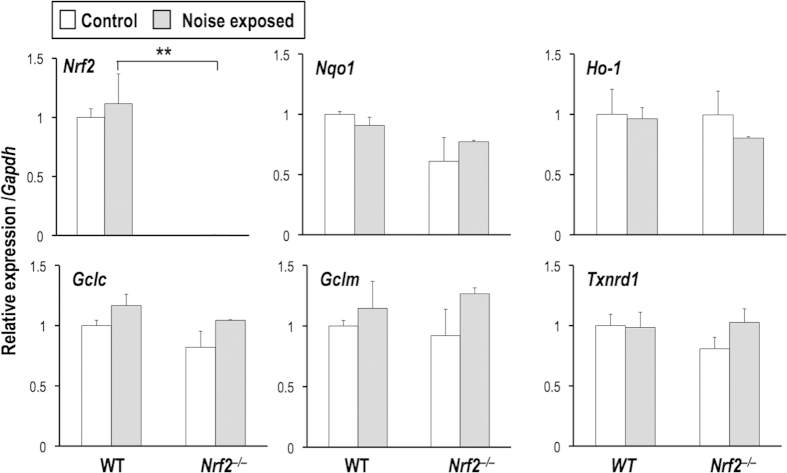
Expression levels of NRF2 target genes in the whole cochlea with or without noise exposure. Expression levels of *Nrf2* and NRF2 target genes (*Nqo1, Ho-1, Gclc, Gclm,* and *Txnrd1*) in the whole cochlea at 4 hr after noise exposure were examined by real-time RT-PCR. All of the samples were quantified against the same standard curve, and each expression level was normalized to *Gapdh* expression. **P < 0.01. Unpaired two-tailed Student’s *t*-test was applied. The data represent the mean ± s.d. (n = 3).

We exposed wild-type and *Nrf2*^–/–^ mice to 96-dB SPL noise for 2 hr and sacrificed them at 4 hr post-exposure for the purification of total RNA from the whole cochlea. Contrary to our expectation, expression of the NRF2 target genes *NAD(P)H:quinone oxidoreductase 1* (*Nqo1*), *heme oxygenase 1* (*Ho-1*), *glutamate-cysteine ligase, catalytic subunit* (*Gclc*), *glutamate-cysteine ligase, modifier subunit* (*Gclm*) and *thioredoxin reductase 1* (*Txnrd1*) did not differ before and after the noise exposure irrespective of genotype (Fig. 2). Based on previous reports suggesting that noise exposure enhances glutathione synthesis in a specific cell population in the cochlea, for instance the stria vascularis^19^,^20^, it is probable that post-exposure induction of NRF2 target genes occurred in a limited compartment of the cochlea and was thereby undetectable in the whole cochlea. Alternatively, the noise exposure might be a modest inducer of NRF2.

### CDDO-Im attenuates NIHL in an Nrf2-dependent manner

**Figure 3:**
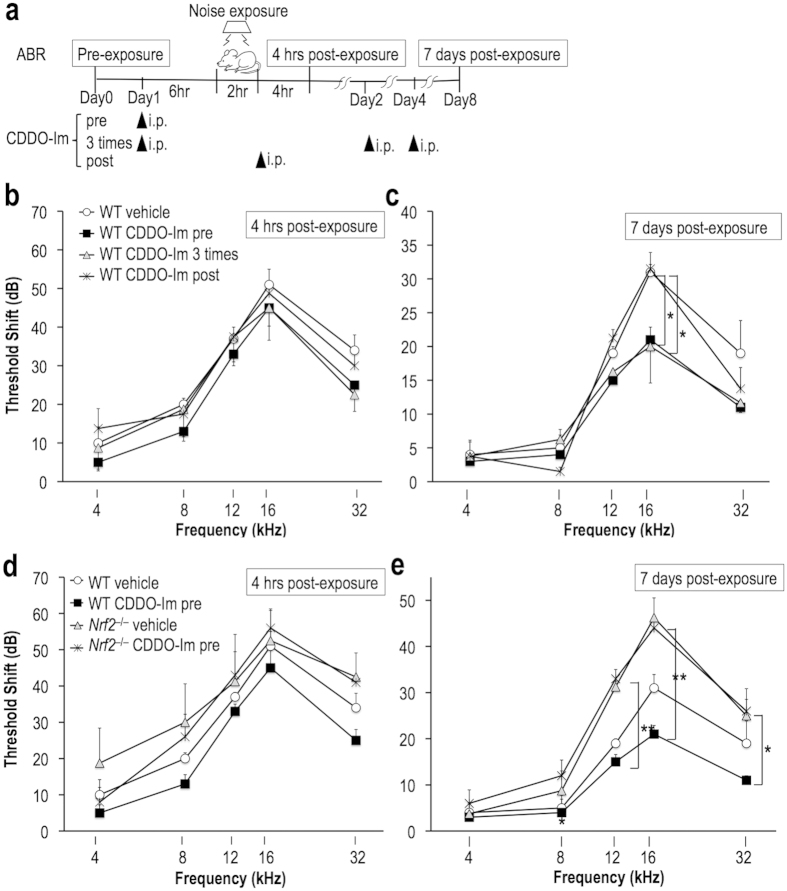
CDDO-Im attenuates NIHL in an NRF2-dependent manner. (**a**) Experimental design for acoustic overexposure and ABR test with three regimens of CDDO-Im. Noise exposure and ABR threshold measurements were conducted as described in the legend to Fig. 1a. The mice in the “pre” group received CDDO-Im 6 hr before noise exposure. Those in the “3 times” group received CDDO-Im 6 hr before, 1 day after, and 3 days after noise exposure. Those in the “post” group received CDDO-Im immediately after noise exposure. (**b,c**) ABR threshold shifts at 4 hr (**b**) and 7 days (**c**) after noise exposure of mice treated with three different regimens of CDDO-Im. The differences between the vehicle-treated and CDDO-Im-treated “pre”/“3 times” groups were statistically significant at 7 days (**c**). (**d,e**) ABR threshold shifts at 4 hr (**d**) and 7 days (**e**) after noise exposure of wild-type and *Nrf2*^*–/–*^ mice treated with the “pre” regimen of CDDO-Im. The differences between the wild-type and *Nrf2*^*–/–*^ mice treated with CDDO-Im were statistically significant at 7 days (**e**). *P < 0.05, **P < 0.01. Analysis of variance followed by Bonferroni *post hoc* test was applied. The data represent the mean ± s.e.m. (n = 5–6). The data obtained from wild-type mice treated with vehicle and those with CDDO-Im pretreatment that are shown in panels **b** and **c** are duplicated in panels **d** and **e** for comparison.

The clear susceptibility of *Nrf2*^–/–^ mice to acoustic hyperexposure combined with the absence of an obvious difference in the expression levels of NRF2 target genes led us to the idea that exogenously induced NRF2 activation would provide further protection from NIHL. We examined the effect of CDDO-Im, which is a triterpenoid possessing a high potency as an NRF2 inducer^16^,^17^,^18^, in protecting the cochlea from the noise. The mice were administered CDDO-Im or vehicle solution and exposed to 96-dB noise for 2 hr according to the regimens shown in Fig. 3a. The ABR thresholds were measured in the same protocol used in the experiments shown in Fig. 1.

We first optimized the conditions of CDDO-Im treatment (Fig. 3a). A group of mice were given a single dose of CDDO-Im before the noise exposure (CDDO-Im pre); another group of mice were given three doses, one before and two after the noise exposure (CDDO-Im 3 times); and still another group of mice were given a single dose right after the noise exposure (CDDO-Im post). The TTSs of the three CDDO-Im-treated groups were not different from that of the vehicle-treated group (Fig. 3b), whereas the PTS of the “CDDO-Im pre” group and the “CDDO-Im 3 times” group was significantly reduced compared with that of the vehicle-treated group (Fig. 3c). Of note, the PTS of the “CDDO-Im post” group was almost exactly the same as that of the vehicle-treated group, indicating that CDDO-Im treatment after noise exposure is ineffective for the suppression of NIHL development.

To verify the NRF2 dependency of the protective effect of CDDO-Im, we treated wild-type and *Nrf2*^–/–^ mice with CDDO-Im according to the “CDDO-Im pre” protocol. The TTS did not significantly differ between the two genotypes (Fig. 3d), while the PTS was significantly elevated in the *Nrf2*^–/–^ mice irrespective of the CDDO-Im treatment applied (Fig. 3e), indicating that CDDO-Im is ineffective in *Nrf2*^–/–^ mice even if it is administered before the noise exposure. Thus, NRF2 is required for the efficacy of CDDO-Im, and pretreatment with CDDO-Im before the noise exposure is critical for the prevention of NIHL.

### NRF2 protects cochlea from noise-induced injury

**Figure 4:**
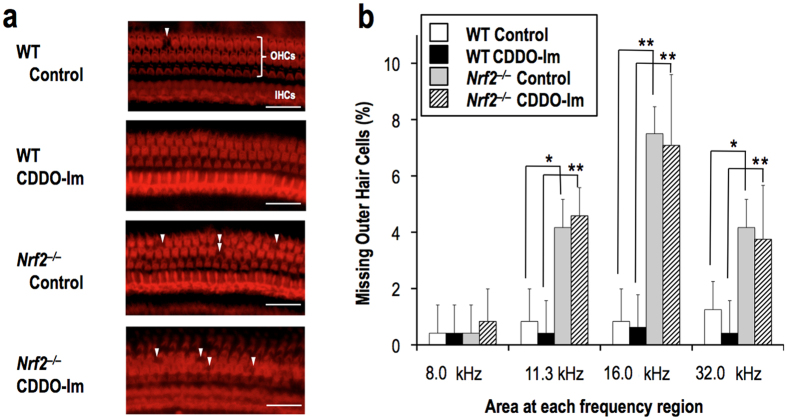
CDDO-Im protects cochlear hair cells from noise-induced damage. (**a**) Surface preparation images of the organ of Corti from wild-type and *Nrf2*^*–/–*^ mice, which were sacrificed after 7 days of noise exposure. CDDO-Im was administered 6 hr before the exposure. Damaged outer hair cells are indicated with arrowheads. All of the experiments were performed at least three times. OHCs, outer hair cells; IHCs, inner hair cells. The scale bar corresponds to 40 μm. (**b**) Quantitative analysis of the damaged outer hair cells in each 400-μm length of the four frequency-specific regions (8.0, 11.3, 16.0, and 32.0 kHz) of the cochlea. *P < 0.05, **P < 0.01. Analysis of variance followed by Bonferroni *post hoc* test was applied. The data represent the mean ± s.e.m. (n = 4).

To clarify the morphological basis of the CDDO-Im-mediated protection, we performed histological analysis of hair cells in the organ of Corti. In surface preparation images, we found the occasional loss of hair cells in the vehicle-treated wild-type mice, whereas missing hair cells were hardly found in the CDDO-Im-treated wild-type mice (Fig. 4a, upper two panels). In contrast, substantial numbers of hair cells were lost in the *Nrf2*^–/–^ mice regardless of the CDDO-Im treatment applied (Fig. 4a, lower two panels). To quantify the histological changes, we counted the damaged outer hair cells in each of four frequency-specific regions (8.0, 11.3, 16.0, and 32.0 kHz) of the cochlea (Fig. 4b). Irrespective of the CDDO-Im treatment, fewer damaged hair cells were observed in the wild-type mice compared with the *Nrf2*^–/–^ mice in all the frequency-specific regions except for the 8.0 kHz region. CDDO-Im treatment tended to reduce the number of damaged hair cells in the wild-type mice, although it did not reach statistical significance. Because the NIHL in the wild-type mice was rather mild irrespective of the CDDO-Im treatment, it was expected that the hair cell loss would not be obvious, based on a previous report that a noise-induced PTS of less than 40 dB is not necessarily accompanied by hair cell loss but results from stereocilia damage alone^21^,^22^. In the *Nrf2*^–/–^ mice, CDDO-Im treatment did not have any effect on the cell number decrease. These results indicate that NRF2 prevents the loss of hair cells in the cochlea.

### CDDO-Im upregulates NRF2 target genes in the cochlea

**Figure 5:**
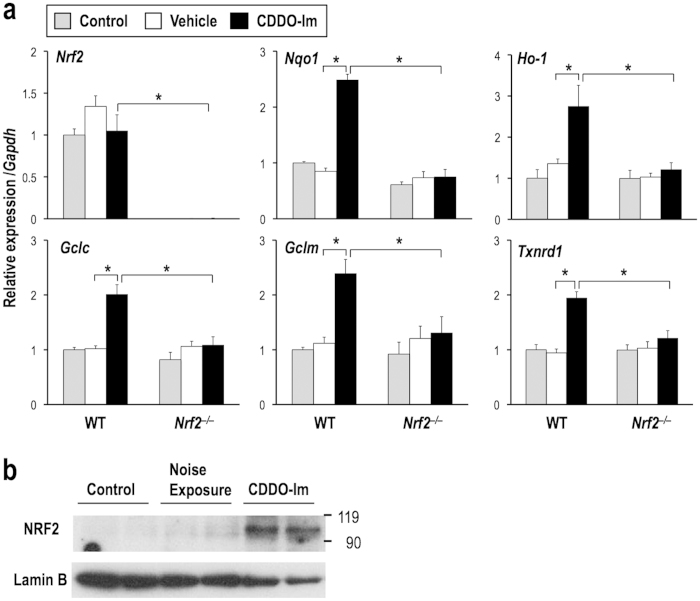
CDDO-Im upregulates the expression of NRF2 target genes in the whole cochlea in an NRF2-dependent manner. (**a**) Expression levels of *Nrf2* and NRF2 target genes (*Nqo1, Ho-1, Gclc, Gclm,* and *Txnrd1*) in the whole cochlea 6 hr after CDDO-Im administration. All of the samples were quantified against the same standard curve, and each expression level was normalized to *Gapdh* expression. *P < 0.05. Analysis of variance followed by Bonferroni *post hoc* test was applied. The data represent the mean ± s.d. (n = 3). (**b**) Immunoblot analysis of NRF2 in the whole cochlea at 4 hr after noise exposure or 6 hr after CDDO-Im administration. Lamin B was detected as a loading control. Full-length blots are presented in Supplementary Figure S1.

To confirm the effect of CDDO-Im, which was intraperitoneally administered, on cochlear protection, we examined the induction of NRF2 target genes in the cochlea. Expression levels of NRF2 target genes (*Nqo1, Ho-1, Gclc, Gclm,* and *Txnrd1*) were examined in the whole cochlea at 6 hr after CDDO-Im administration. CDDO-Im significantly upregulated the NRF2 target genes in the wild-type mice but not in the *Nrf2*^–/–^ mice (Fig. 5a). To verify the accumulation of NRF2, we conducted immunoblot analysis of NRF2 using the cochlear lysates (Fig. 5b). Consistent with the expression levels of NRF2 target genes (Figs 2 and 5a), the protein levels of NRF2 were not elevated by the simple noise exposure but were elevated in the cochlea after the CDDO-Im administration. Thus, intraperitoneally administered CDDO-Im was effective in activating NRF2 to increase the expression of NRF2 target genes in the cochlea.

### CDDO-Im reduces post-exposure oxidative stress in the cochlea

**Figure 6:**
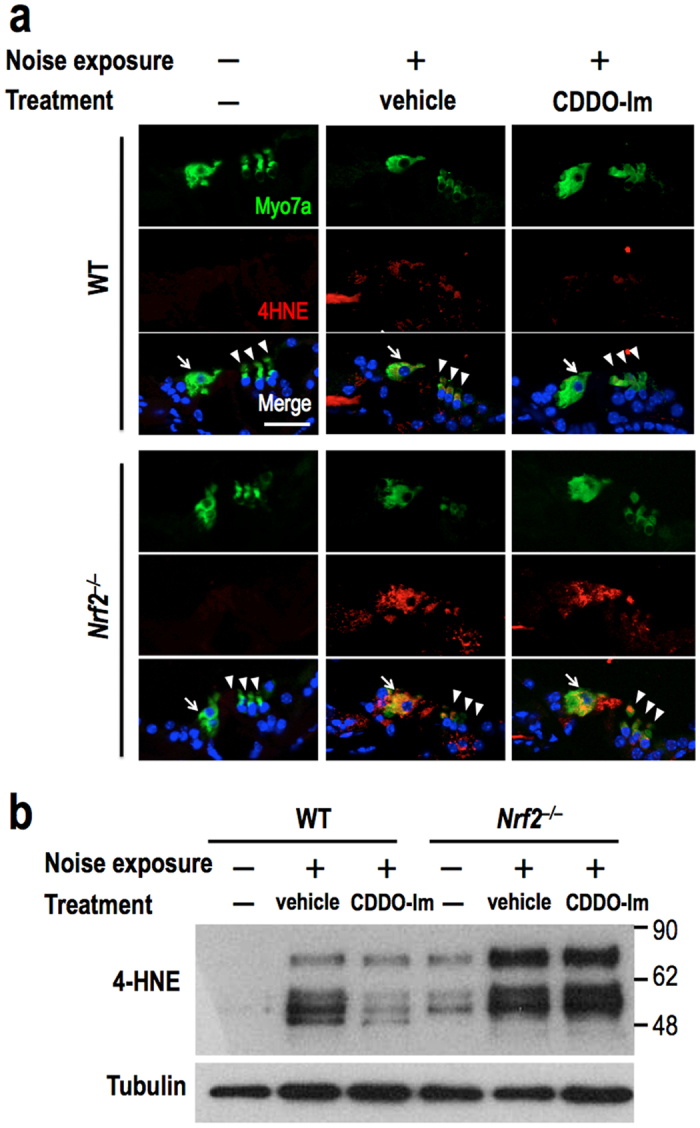
Noise-induced lipid peroxidation in the cochlea is suppressed by CDDO-Im administration in an NRF2-dependent manner. CDDO-Im or vehicle was administered to the mice 6 hr before the noise exposure, and the mice were sacrificed at 4 hr post-exposure. (**a**) Immunofluorescence detection of 4HNE in the organ of Corti of wild-type and *Nrf2*^*–/–*^ mice. All of the experiments were performed at least three times. Myosin 7a (Myo7a) is a marker of hair cells (green). The nuclei were counter-stained with DAPI (blue). 4HNE signals are shown in red. Inner hair cells and outer hair cells are indicated with arrows and arrowheads, respectively. The scale bar corresponds to 40 μm. (**b**) Immunoblot analysis of 4HNE accumulation in the whole cochlea. Tubulin was detected as a loading control. Full-length blots are presented in Supplementary Figure S2.

To evaluate an impact of CDDO-Im treatment on the level of oxidative stress induced by the noise exposure, we exploited 4-hydroxy-2-nonenal (4HNE) as an indicator of oxidative stress and detected 4HNE adducts in the organ of Corti by immunofluorescence (Fig. 6a). Mice were pretreated with or without CDDO-Im at 6 hr before the start of the noise exposure and sacrificed 4 hr after the end of the noise exposure.

Inner and outer hair cells, which were marked by positive staining with an anti-myosin 7a antibody, gave no detectable 4HNE signals in the absence of the noise exposure, irrespective of genotype. Upon noise exposure, the wild-type hair cells accumulated 4HNE adducts. This accumulation was decreased by the administration of CDDO-Im. In contrast, the *Nrf2*^–/–^ hair cells accumulated dramatically high levels of 4HNE adducts, against which CDDO-Im was ineffective. Immunoblot detection of 4HNE adducts in the whole cochlea produced results consistent with the immunofluorescence data (Fig. 6b). The *Nrf2*^–/–^ cochleas exhibited abundant accumulation of 4HNE adducts after noise exposure, and this accumulation was not altered by CDDO-Im treatment. These results indicate that NRF2 deficiency exacerbates noise-induced oxidative damage of the cochlea and that CDDO-Im reduces cochlear oxidative stress in an NRF2-dependent manner. Thus, NRF2 protects the cochlea against acoustic overexposure by reducing oxidative stress. Of note, anti-4HNE antibody yielded three or four discrete bands ranging from 50 to 70 kDa instead of smears ranging over wide molecular weights, which was also observed in previous reports^23^,^24^,^25^. This result suggest that specific proteins, which are yet unidentified, have a greater chance of being modified by 4HNE than others.

### NRF2-deficient cochlea exhibits significant decrease of reduced glutathione (GSH)

**Figure 7:**
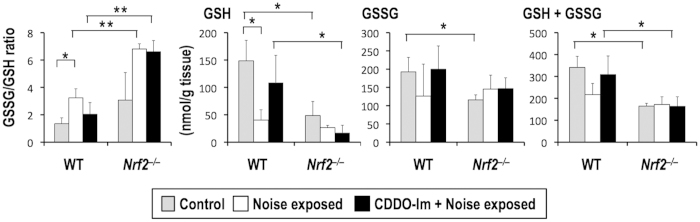
Quantification of glutathione in cochleas with and without noise exposure. CDDO-Im or vehicle was administered to the mice 6 hr before the noise exposure, and the mice were sacrificed at 4 hr post-exposure. Reduced and oxidized glutathione and their ratios are shown. *P < 0.05, **P < 0.01. Unpaired two-tailed Student’s *t*-test was applied. The data represent the mean ± s.d. (n = 3).

To characterize the effect of the noise-induced oxidative stress on cochlea, we quantified reduced (GSH) and oxidized (GSSG) forms of glutathione (Fig. 7). We divided wild-type mice and *Nrf2*^–/–^ mice into three groups; without treatment, with noise exposure, and with CDDO-Im pre-treatment and noise exposure. Following the same protocol employed for the histological analysis, we collected inner ears and processed them for the glutathione quantification.

Basal levels of GSH and GSSG were significantly lower in the *Nrf2*^–/–^ inner ears than those of wild-type mice, indicating that NRF2 contributes to the maintenance of the total glutathione level and its redox balance even under conditions without any treatments. After the noise exposure, GSH levels were decreased in both wild-type and *Nrf2*^–/–^ inner ears, resulting in the increase of GSSG/GSH ratios. These results are consistent with the increased levels of 4HNE adducts, which is an oxidative stress indicator (see Fig. 6), verifying the accumulation of oxidative stress after the noise exposure, particularly in the *Nrf2*^–/–^ inner ears. Importantly, administration of CDDO-Im prior to the noise exposure prevented the GSH decrease in wild-type inner ears, which was not observed in *Nrf2*^–/–^ inner ears. These results are explained by the major contribution of NRF2 to glutathione reduction and glutathione synthesis. Therefore, pre-activation of NRF2 increases the GSH level, a parameter of cellular anti-oxidant capacity, achieving the effective protection of cochlea from noise-induced oxidative damage.

### An SNP in the human NRF2 promoter is associated with susceptibility to SNHL

**Figure 8:**
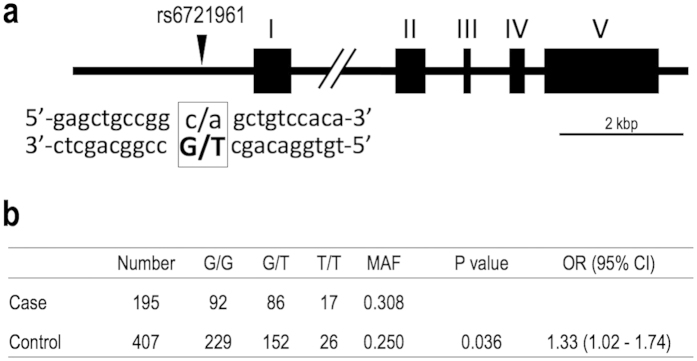
An SNP in the promoter of the human *NRF2* gene is associated with susceptibility to SNHL. (**a**) Schematic illustration of the SNP (rs6721961) in the promoter of the human *NRF2* gene. (**b**) Frequency of the SNP genotypes in JSDF members with (case) or without (control) an elevation of the hearing threshold at 4 kHz. *P* value was calculated by trend test. MAF, minor allele frequency; OR, odds ratio; CI, confidence interval.

Finally, we investigated the impact of NRF2 function on human hearing loss. A common *NRF2* SNP (rs6721961) located in the promoter region of the human *NRF2* gene (Fig. 8a) has been shown to be correlated with the transcription level of the *NRF2* gene^26^,^27^. The “G” and “T” alleles result in higher and lower expression of NRF2 mRNA, respectively, and possession of the former is beneficial for protection from various pathological conditions^27^,^28^,^29^. We analyzed the association between rs6721961 genotype and hearing level using data taken from the health examination of Japan Self-Defense Force (JSDF) personnel. The JSDF members are regarded as a relatively homogeneous population who are subjected to occupational noise exposure^30^. Genotyping of the *NRF2* SNP in 602 participants demonstrated that the frequency of the “T” allele was 30.8% and 25.0% in the participants with and without an elevation of the hearing threshold at 4 kHz (195 cases and 407 controls in Fig. 8b), respectively. A trend test across the genotype categories revealed that the *NRF2* SNP is associated with the elevation of the hearing threshold at 4 kHz (P = 0.036; Fig. 8b). The P value for Hardy-Weinberg equilibrium was 0.68 in the control group, suggesting no evidence of systematic typing errors. The odds ratio was 1.33 (95% confidence interval: 1.02–1.74), which indicates that the “T” allele of rs6721961 significantly increases susceptibility to the elevation of the hearing threshold at 4 kHz. In contrast, there were no significant differences (P = 0.322) in the frequency of the “T” allele between those with and without an elevation of the hearing threshold when the average hearing level at high frequencies (2, 4, and 8 kHz) was used for the criterion instead of that at 4 kHz. Thus, possession of the “T” allele significantly elevates the probability of having a 4-kHz notch in the audiogram, which is considered the signature configuration for NIHL^31^.

## Discussion

The noise exposure experiments performed using *Nrf2*^–/–^ mice in this study revealed the critical contribution of NRF2 to cochlear protection from noise-induced damage. A human SNP that reduces the transcription level of the *NRF2* gene was significantly associated with impaired hearing levels in a cohort of JSDF members subjected to occupational noise exposure, which strongly supports the notion that higher NRF2 activity is beneficial for cochlear protection from the oxidative damage induced by excessive noise. An important finding of this study is that NRF2 activation before the noise exposure was necessary to obtain significant hearing improvement, indicating that sufficient antioxidant capacity at the moment of noise exposure is critical for suppressing the development of a PTS and NIHL afterwards. Therefore, NRF2 activation is best utilized for the prevention of NIHL, particularly for scheduled noise exposure.

Several compounds have been reported to protect the inner ear from NIHL by enhancing antioxidant capacity^3^, some of which are downstream products of the KEAP1-NRF2 system or their mimetics, such as GSH, glutathione monoethylester (GSHE) and N-acetylcysteine (NAC). Other compounds effective for NIHL improvement have been shown to activate NRF2, including ebselen^32^, resveratrol^33^, allopurinol^34^, and deferoxamine mesylate^35^. Recent studies have suggested that rosmarinic acid is effective at reducing noise-induced cochlear damage by activating the NRF2 pathway^25^. Consistent with these reports, our current study has provided conclusive evidence of the essential role of NRF2 in cochlear protection by examining *Nrf2*^–/–^ mice and also provided mechanistic insight into the modes of action of these compounds.

Like NIHL, SNHL caused by ototoxic drugs such as aminoglycosides and cisplatin has also been suggested to be alleviated by the administration of NRF2-activating drugs^36^,^37^. This is expected considering that many detoxifying enzymes are robustly induced by NRF2. Another class of SNHL is AHL, which is the most common class of SNHL in adult populations and for which oxidative damage of the cochlea has been considered the most critical cause^38^. Mitochondria are a major source of endogenous ROS, and in the course of aging, progressive decline of mitochondrial function results in the increased production of ROS, eliciting oxidative damage and dysfunction of various tissues^39^. Caloric restriction has been shown to be effective for AHL prevention by enhancing NADPH production in mitochondria, replenishing a sufficient amount of reduced glutathione and reducing mitochondria-derived ROS^40^. It is plausible that NRF2 activation is effective for the prevention of AHL via the induction of antioxidant proteins, resulting in the reduction of ROS levels.

Although NRF2 activity is primarily regulated through protein stability by KEAP1-dependent ubiquitination, the transcription level of the *NRF2* gene provides another layer of regulation of NRF2 activity. Indeed, transcriptional regulation of *NRF2* has been shown to impact the susceptibility to various pathological conditions in mice and humans^27^,^28^,^29^,^41^. In humans, the *NRF2* promoter SNP, which was genotyped in this study, influences the *NRF2* transcription level, and individuals that possess the SNP that lowers *NRF2* expression have been found to be more susceptible to acute lung injury and smoking-related lung cancer^27^,^29^. In the current study, we demonstrated a significant association between the SNP and the susceptibility to an elevation of the hearing threshold at 4 kHz (audiometric 4-kHz notch) in a cohort of JSDF personnel, who are constantly exposed to occupational noise^30^. Considering the broad contribution of NRF2 to oxidative stress-related pathogenesis, this result suggests that oxidative stress is indeed closely related to NIHL pathogenesis and that lower antioxidant capacity due to lower expression of the *NRF2* gene is a risk factor for NIHL. The administration of CDDO-Im stabilizes NRF2 and increases NRF2 protein levels, which should successfully overcome the low basal antioxidant capacity due to the SNP genotype. We propose pretreatment with NRF2-activating drugs as a beneficial strategy for the prevention of NIHL in people who are frequently exposed to intense noise, particularly for those possessing the low expressor allele of *NRF2*.

## Methods

### Animals

Wild-type and Nrf2 knock out (*Nrf2*^*–/–*^) mice with a C57BL/6 (Jackson Laboratories) genetic background were used in this study (6–7 weeks of age, weighing 17–20 g at study onset). Mice were genotyped by PCR, using the following primers: Nrf2 forward 5′-TGG ACG GGA CTA TTG AAG GCT G-3′, Nrf2–/– reverse 5′-GCG GAT TGA CCG TAA TGG GAT AGG-3′, and Nrf2 + / + reverse 5′-GCC GCC TTT TCA GTA GAT GGA GG-3′. Throughout the experiment, the mice were housed under a constant temperature with food and water available *ad libitum.* All mice were treated in accordance with guidelines presented in *The Standards for Human Care and Use of Laboratory Animals of Tohoku University* and *Guidelines for Proper Conduct of Animal Experiments* by the Ministry of Education, Culture, Sports, Science, and Technology of Japan. All the animal experiments were approved by the Ethics Committee for Animal Experiments of Tohoku University Graduate School of Medicine.

### CDDO-Im treatment

Mice were intraperitoneally administered vehicle (DMSO) or CDDO-Im (30 μmol/kg body weight, obtained from Mochida Pharmaceuticals Co., Ltd., Tokyo, Japan). In the ABR study, CDDO-Im was given to the mice according to the regimen shown in Fig. 3a. For the quantitative RT-PCR, immunohistochemistry, immunoblot analysis, and glutathione measurement, the mice received CDDO-Im at 6 hr pre-exposure or at 6 hr pre-sacrifice.

### Acoustic overexposure

Mice were kept awake and unrestrained in an anechoic chamber and were exposed to an 8–16 kHz octave-band noise at 96 dB sound pressure level (SPL) for 2 hr. The sound was generated by a waveform generator (SF-06, Random Noise Generator; RION), amplified by an audio amplifier (D-75A; Crown), and filtered by an audio filter (Multifunction Filter; NF Corporation). The sound was presented in an open field by a dome tweeter (2446H; JBL) positioned at the center of the cage. Sound level was measured using a sound level meter (2250L; Brüel & Kjær.)

### Hearing function test

Mice were anesthetized with ketamine (100 mg/kg body weight) and xylazine (20 mg/kg body weight) by intraperitoneal administration. ABR recordings were performed using a TDT System 3 auditory-evoked potential workstation and BioSigRP software (Tucker-Davis Technologies). ABR responses were evoked using bursts of pure tones at frequencies of 4, 8, 12, 16, and 32 kHz. Evoked responses were averaged across 1,000 sweeps. Responses were collected for stimulus levels in 5-dB steps from 100 dB SPL to 10 dB SPL. The ABR threshold was defined as the lowest sound intensity sufficient to elicit at least one peak in the averaged ABR.

### Real time quantitative RT-PCR

Mice were deeply anesthetized and transcardially perfused with ice-cold 10 mM phosphate-buffered saline (PBS) (pH 7.4). The cochleas were quickly removed from the skull, collected on ice, and stored at –80 °C in RNAlater (Life Technologies). The cochleas were homogenized in ISOGEN (Nippon gene), and for each sample, total RNA was purified from two whole cochleas. cDNA was synthesized using reverse transcriptase (ReverTra Ace, Toyobo). Quantitative PCR was performed on an Applied Biosystems 7300 sequence detector system with Power SYBR Green PCR Master Mix (Applied Biosystems), THUNDERBIRD SYBR qPCR Mix (Toyobo) for the SYBR Green system and THUNDERBIRD Probe qPCR Mix (Toyobo) for the Taqman probe system. The amplification reaction mixture (25μl) contained 400 nM of each primer in the SYBR Green system, or 300 nM of each primer and 200 nM of a probe in the Taqman probe system. A thermal cycling condition used in this study is as follows; 2 min at 50 °C and 10 min at 95 °C followed by 50 cycles of 95 °C for 15 s and 60 °C for 60 s. The primers utilized in this study are shown in Supplementary Table S1.

### Morphological analysis of hair cells

Mice were deeply anesthetized and transcardially perfused with 10 mM PBS and 4% paraformaldehyde (PFA) in 10 mM PBS (pH 7.4). The inner ears were quickly removed from the skull and immediately placed in 4% PFA. Small openings were made at the round window, oval window, and apex of the cochlea. The inner ears were fixed with 4% PFA at 4 °C overnight and then decalcified in 10% EDTA for 2 days at 4 °C. For hair cell counting, surface preparations of the basilar membrane and the organ of Corti were processed. The hair cells were stained for F-actin with rhodamine-conjugated phalloidin (1:100, Invitrogen) for 1 hr at room temperature under light-protected conditions. High-power fluorescence images were obtained using a microscope (BZ-9000, Keyence) and BZ-H1 software (Keyence), and the full-length cochlea was assembled and analyzed in Photoshop CS4 (Adobe). ImageJ software (NIH) was used to assemble and analyze the computed frequency map. Damaged and undamaged outer hair cells were counted in each 400-μm length of the four frequency-specific regions (8.0, 11.3, 16.0, and 32.0 kHz) of the cochlea.

### Immunohistochemistry

Cochlear samples were prepared using a procedure similar to that used for the histological hair cell analysis. The decalcified samples of cochlea were rinsed in 10 mM PBS containing 10% and 30% sucrose, embedded in Tissue-Tek O.T.C. Compound (Sakura Finetechnical), and frozen in liquid nitrogen. Thin sections (8 μm) were obtained with a cryostat (CM3000, Leica Instruments). Immunohistochemistry was performed according to a previously described procedure with modification^42^. Briefly, tissue sections were rinsed in 0.1% Triton X-100/Tris buffered saline (TBS), blocked with 3% bovine serum albumin/0.3% Triton X-100/TBS (TBST) for 30 min at room temperature, and incubated with an unconjugated AffiniPure Fab fragment anti-mouse IgG (1:10, Jackson ImmunoResearch) for 2 hr at room temperature. The sections were incubated overnight with primary antibodies (anti-4HNE; 1:400, JaICA. anti-Myosin 7a; 1:500, Abcam) diluted in 0.3% TBST at 4 °C. All of the sections were washed three times in 0.1% TBST and incubated with secondary antibodies (Cy3 anti-mouse; 1:500, Jackson ImmunoResearch, Alexa Fluor 488 anti-rabbit; 1:500, Jackson ImmunoResearch) for 1 hr at room temperature under light-protected conditions. The nuclei were counterstained with 4′,6-diamidino-2-phenylindole (DAPI) (1:2,000).

### Equipment and settings

Fluorescence images were obtained using a laser-scanning confocal microscope (LSM 780, Zeiss) equipped with a Plan-Neofluar 40x/1.3 oil immersion objective lens and Blue-diode 405 nm, Multi-Argon 458/488/514 nm and DPSS 561 nm lasers. The images were analyzed using ZEN software (Zeiss).

### Immunoblot analysis

Mice were deeply anesthetized and transcardially perfused with ice-cold 10 mM PBS (pH 7.4). The cochleas were quickly removed from the skull, collected on ice and stored at –80 °C in 10 mM PBS (pH 7.4). As described previously^24^, both cochleas of each mouse were homogenized in 400 μl of RIPA buffer [10 mM Tris-HCl (pH 7.4), 150 mM NaCl, 0.1% sodium deoxycholate, 0.1% SDS, 1% Nonidet P-40, 1 mM dithiothreitol, and 1x complete protease inhibitor cocktail tablets (Roche)] for 15 s. After centrifugation for 15 min at 12,000 rpm at 4 °C, 250 μl of the supernatant was collected and 6x SDS sample buffer was added. The solution was then boiled at 95 °C for 5 min. Each sample was separated by SDS-PAGE on a 10% gel. Proteins were then transferred onto PVDF membranes (Millipore). After blocking with 5% skim milk in 0.05% Tween 20/TBS, the membranes were incubated overnight at 4 °C with an anti-4HNE antibody (1:100, JaICA) or anti-NRF2 antibody (1:100)^43^. After rinsing in 0.05% Tween 20/TBS, the membranes were incubated for 30 min at room temperature with a horseradish peroxidase-conjugated anti-mouse IgG antibody (1:2,000, Life Technology) for 4HNE or anti-rat IgG antibody (1:1,000) for NRF2. The immunoreactive bands were detected with Chemi-Lumi One L (Nacalai Tesque). Equal protein loading among individual lanes was confirmed by reprobing the membrane with an anti-Tubulin mouse monoclonal antibody (1:2,000, Sigma Aldrich) or anti-Lamin B goat polyclonal antibody (1:1,000, Santa Cruz Biotech).

### Measurement of glutathione in the mouse cochlea

Both temporal bones (including the cochlea and vestibule, approximately 30 mg) of each sample were dissected 4 hr after the noise exposure and snap-frozen in liquid nitrogen. The frozen tissue was homogenized in 500 μL of cold methanol containing 50 μM internal standard solution (Human Metabolome Technologies, Tsuruoka, Japan). The homogenate was mixed with 500 μL chloroform and 200 μL Milli-Q water and further homogenized and centrifuged at 750 g for 10 min at 4 °C. The aqueous layer was collected by filtration through a Millipore 5-kDa cutoff filter and centrifuged at 9,100 g to remove proteins. The filtrate was lyophilized, suspended in 25 μL Milli-Q water and analyzed using CE-TOFMS (Human Metabolome Technologies, Tsuruoka, Japan).

CE-TOFMS was carried out on an Agilent CE Capillary Electrophoresis System equipped with an Agilent 6210 time-of-flight mass spectrometer, an Agilent 1100 isocratic HPLC pump, an Agilent G1603A CE-MS adapter kit and an Agilent G1607A CE-ESI-MS sprayer kit (Agilent Technologies, Santa Clara, CA). The system was controlled by Agilent G2201AA Chem- Station software version B.03.01 for CE (Agilent Technologies, Santa Clara, CA).

The separation and detection of the cationic metabolites were carried out as described previously^44^. A fused silica capillary (50 lm i.d.; 980 cm total length) with cation buffer solution (Human Metabolome Technologies, Tsuruoka, Japan) was used. The sample was injected at a pressure of 50 mbar for 10 s (approximately 10 nL). The applied voltage was set at 27 kV. Electrospray ionization-mass spectrometry (ESI-MS) was conducted in positive ion mode, and the capillary voltage was set at 4,000 V. The spectrometer was scanned from m/z 50 to 1,000.

The raw data that were obtained using CE-TOFMS were processed with Keio MasterHands, which was developed by Keio University^45^. Signal peaks corresponding to GSH and GSSG were extracted. GSH and GSSG migration times (MTs) were normalized using those of the internal standards. The resultant relative area values were further normalized to the sample amount.

### Association study of an NRF2 promoter SNP and susceptibility to SNHL

For the association analysis between the SNP in the *NRF2* promoter, rs6721961, and SNHL susceptibility, 602 Japanese males were recruited from health examinations conducted at Self-Defense Forces Central Hospital. We only extracted the examination data of the 49- and 50-year-old participants to exclude the effects of age-related hearing loss. This study protocol was approved by the Ethics Committee of the National Defense Medical College and Self-Defense Forces Central Hospital. Written informed consents were obtained from all of the participants, and all of the investigations were conducted according to the principles expressed in the Declaration of Helsinki. Pure-tone audiometry was conducted with an audiometer (AA-79S, RION Inc., Tokyo, Japan). To determine the precise impact of the SNP on SNHL, participants with conductive hearing loss were excluded through careful examination by two independent otolaryngologists before the data analysis (I.M. and K.M.). These participants were divided into controls (hearing level at 4 kHz of the better side ear ≤20 dB hearing level (HL)) and cases (>20 dB HL). Genomic DNA was extracted from whole peripheral blood cells^46^. Genotyping of the *NRF2* promoter SNP, rs6721961, was performed using the TaqMan method (Life Technologies Corporation, Carlsbad, CA USA) with a LightCycler 480 (Roche Diagnostics, Mannheim, Germany)^43^. The association between *NRF2* and SNHL was examined using the Cochran-Armitage trend test by the software R (version 3.1.1) (http://www.r-project.org/).

### Statistical analysis

Quantitative data are presented as the mean ± s.e.m. or s.d. Student’s *t*-test was utilized to compare the two groups. For studies employing multiple testing, we used one- or two-way analysis of variance (ANOVA) followed by Bonferroni’s *post hoc* test. For all tests, P values of <0.05 were considered statistically significant.

## Additional Information

**How to cite this article**: Honkura, Y. *et al*. NRF2 Is a Key Target for Prevention of Noise-Induced Hearing Loss by Reducing Oxidative Damage of Cochlea. *Sci. Rep.* **6**, 19329; doi: 10.1038/srep19329 (2016).

## Supplementary Material

Supplementary Information
