# Supplementary material for: NRF2 Is a Key Target for Prevention of Noise-Induced Hearing Loss by Reducing Oxidative Damage of Cochlea
Source: Sci Rep. 2016 Jan 18;6:19329. doi: 10.1038/srep19329 (PMC4726010; doi:10.1038/srep19329)

NRF2 Is a Key Target for Prevention of Noise-Induced Hearing Loss by Reducing Oxidative Damage of Cochlea

Yohei Honkura[1,2], Hirotaka Matsuo[3,*], Shohei Murakami[2], Masayuki Sakiyama[3], Kunio Mizutari[4], Akihiro Shiotani[4], Masayuki Yamamoto[5], Ichiro Morita[6], Nariyoshi Shinomiya[3], Tetsuaki Kawase[1], Yukio Katori[1], Hozumi Motohashi[2,*]

[1]Department of Otolaryngology-Head & Neck Surgery, Tohoku University Graduate School of Medicine, 1-1 Seiryo-machi, Aoba-ku, Sendai, 980-8574, Japan.
[2]Department of Gene Expression Regulation, Institute of Development, Aging and Cancer, Tohoku University, 4-1 Seiryo-machi, Aoba-ku, Sendai, 980-8575, Japan.
[3]Department Integrative Physiology and Bio-Nano Medicine, National Defense Medical College, 3-2 Namiki, Tokorozawa, Saitama 359-8513, Japan.
[4]Department of Otolaryngology-Head and Neck Surgery, National Defense Medical College, 3-2 Namiki, Tokorozawa, Saitama 359-8513, Japan.
[5]Department of Medical Biochemistry, Tohoku University Graduate School of Medicine, 2-1 Seiryo-machi, Aoba-ku, Sendai, 980-8575, Japan.
[6]Department of Otolaryngology-Head and Neck Surgery, Self-Defense Forces Central Hospital, 1-2-24, Ikejiri, Setagaya, Tokyo 154-8532, Japan.

*To whom correspondence should be addressed: Hirotaka Matsuo and Hozumi Motohashi

Hirotaka Matsuo
Department of Integrative Physiology and Bio-Nano Medicine,
National Defense Medical College,
3-2 Namiki, Tokorozawa, Saitama 359-8513, Japan
TEL: 81-4-2995-1482,
FAX: 81-4-2996-5187,
E-MAIL: hmatsuo@ndmc.ac.jp

Hozumi Motohashi
Department of Gene Expression Regulation,
Institute of Development, Aging and Cancer,
Tohoku University,
4-1 Seiryo-machi, Aoba-ku, Sendai, 980-8575, Japan
TEL: 81-22-717-8550,
FAX: 81-22-717-8554,
E-MAIL: hozumi.motohashi.a7@tohoku.ac.jp

SUPPLEMENTARY TABLE

Supplementary Table S1 | Sequences of primers for quantitative real-time PCR analysis.

Gene	Forward primer	Reverse primer	Probe
Gapdh	5’-GAGATGATGAC CCTTTTGGC-3’	5’-GTCGTGGAGTCT ACTGGTGTCTT-3’	-
Nrf2	5’-CAAGACTTGGG CCACTTAAAAGAC-3’	5’-AGTAAGGCTTTC CATCCTCATCAC-3’	5’-FAM-AGGCGGC TCAGCACCTTGTA TCTTGA-TAMRA-3’
Nqo1	5’-AGCTGGAAGCT GCAGACCTG-3’	5’-CCTTTCAGAATG GCTGGCA-3’	5’-FAM-ATTTCAGT TCCCATTGCAGTG GTTTGGG-TAMRA-3’
Ho-1	5’-GTGATGGAGCG TCCACAGC-3’	5’-TTGGTGGCCTCC TTCAAGG-3’	5’-FAM-CGACAGC ATGCCCCAGGATT TGTC-TAMRA-3’
Gclc	5’-ATCTGCAAAGG CGGCAAC-3’	5’-ACTCCTCTGCAG CTGGCTC-3’	5’-FAM-ACGGGTG CAGCAAGGCCCA-TAMRA-3’
Gclm	5’-TGGAGCAGCTGT ATCAGTGG-3’	5’-AAATCTGGTGGC ATCACACA-3’	-
Txnrd1	5’-AGAAAGTGCTG GTCTTGGATTTTG-3’	5’-ACACGTTCCTCC GAGACCC-3’	5’-FAM-TCTGGTCC CAAGAGGAGTCG GTGTG-TAMRA-3’

SUPPLEMENTARY FIGURES

Supplementary Figure S1 | Full-length immunoblot reacted with anti-NRF2 antibody shown in Figure 5b.   .

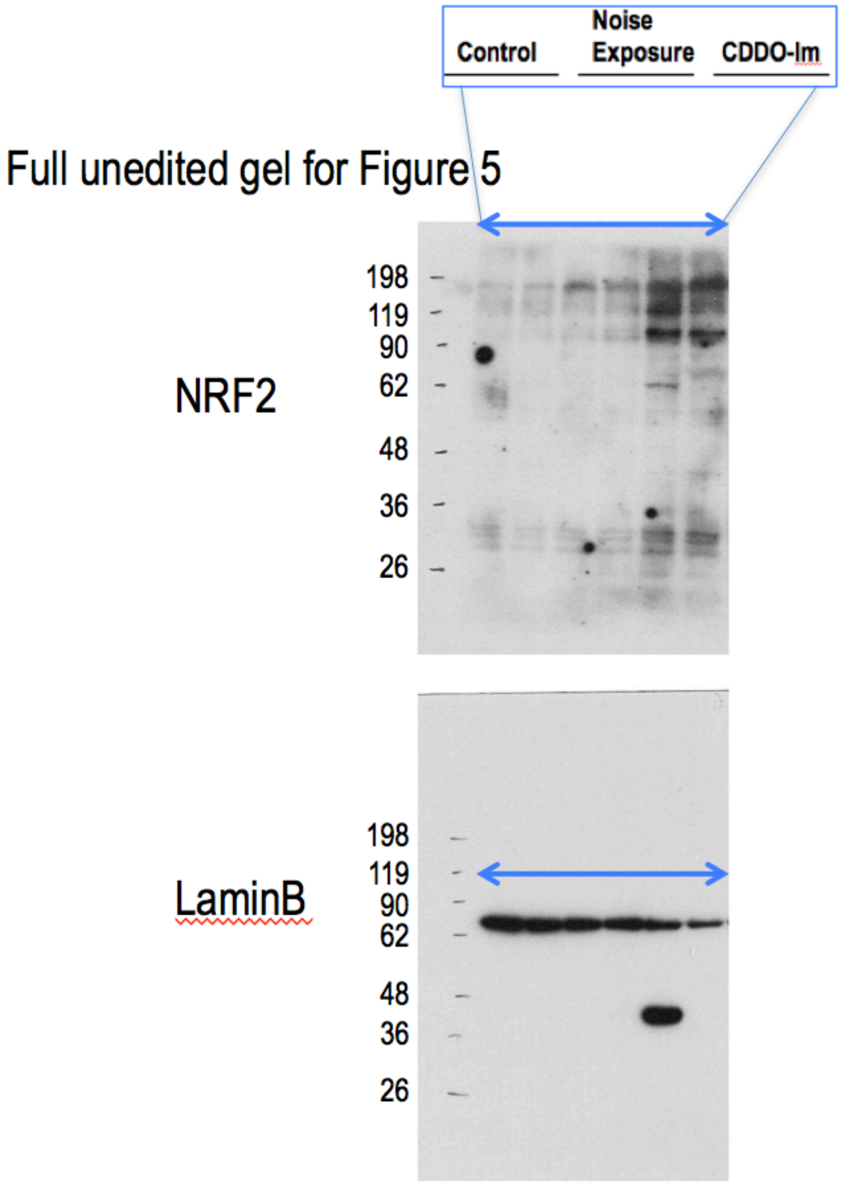

Supplementary Figure S2 | Full-length immunoblot reacted with anti-4-HNE antibody shown in Figure 6b.   .

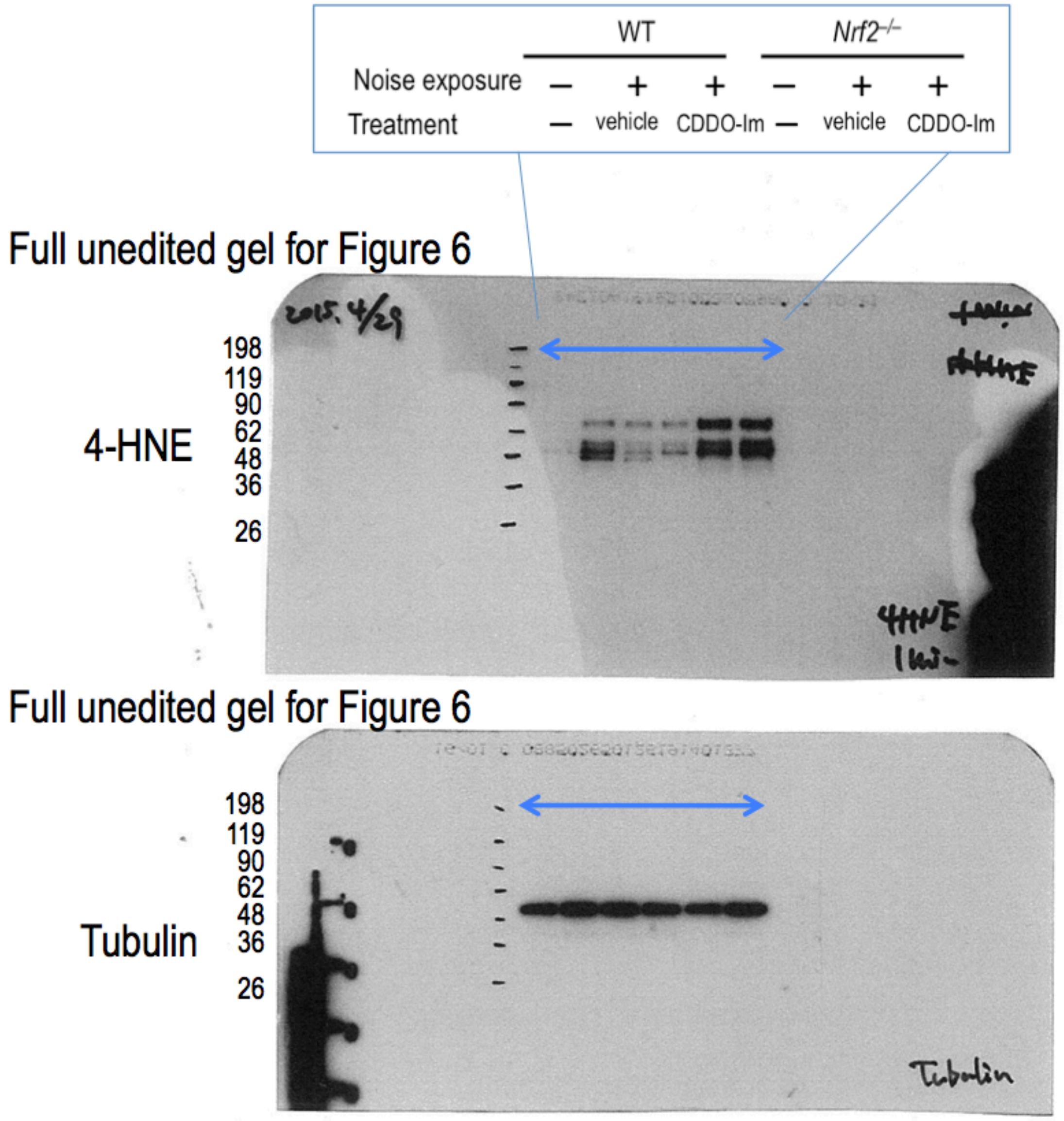

Supplement: Supplementary Information [file srep19329-s1.pdf]
